# Early Cenozoic Decoupling of Climate and Carbonate Compensation Depth Trends

**DOI:** 10.1029/2019PA003601

**Published:** 2019-06-17

**Authors:** S. E. Greene, A. Ridgwell, S. Kirtland Turner, D. N. Schmidt, H. Pälike, E. Thomas, L. K. Greene, B. A. A. Hoogakker

**Affiliations:** ^1^ School of Geography, Earth and Environmental Sciences University of Birmingham Birmingham UK; ^2^ BRIDGE, School of Geographical Sciences University of Bristol Bristol UK; ^3^ Department of Earth Sciences University of California at Riverside Riverside CA USA; ^4^ School of Earth Sciences University of Bristol Bristol UK; ^5^ MARUM‐Center for Marine Environmental Sciences University of Bremen Bremen Germany; ^6^ Department of Geology and Geophysics Yale University New Haven CT USA; ^7^ Department of Earth and Environmental Sciences Wesleyan University Middletown CT USA; ^8^ University Program in Ecology Duke University Durham NC USA; ^9^ Department of Evolutionary Anthropology Duke University Durham NC USA; ^10^ Institute of Life and Earth Sciences Heriot Watt University Edinburgh UK

**Keywords:** carbonate compensation depth, Earth system modeling, weathering feedback

## Abstract

Our understanding of the long‐term evolution of the Earth system is based on the assumption that terrestrial weathering rates should respond to, and hence help regulate, atmospheric CO_2_ and climate. Increased terrestrial weathering requires increased carbonate accumulation in marine sediments, which in turn is expected to result in a long‐term deepening of the carbonate compensation depth (CCD). Here, we critically assess this long‐term relationship between climate and carbon cycling. We generate a record of marine deep‐sea carbonate abundance from selected late Paleocene through early Eocene time slices to reconstruct the position of the CCD. Although our data set allows for a modest CCD deepening, we find no statistically significant change in the CCD despite >3 °C global warming, highlighting the need for additional deep‐sea constraints on carbonate accumulation. Using an Earth system model, we show that the impact of warming and increased weathering on the CCD can be obscured by the opposing influences of ocean circulation patterns and sedimentary respiration of organic matter. From our data synthesis and modeling, we suggest that observations of warming, declining δ^13^C and a relatively stable CCD can be broadly reproduced by mid‐Paleogene increases in volcanic CO_2_ outgassing and weathering. However, remaining data‐model discrepancies hint at missing processes in our model, most likely involving the preservation and burial of organic carbon. Our finding of a decoupling between the CCD and global marine carbonate burial rates means that considerable care is needed in attempting to use the CCD to directly gauge global carbonate burial rates and hence weathering rates.

## Key Points

- Compilation of deep‐sea carbonate preservation shows modest CCD deepening across the long‐term Late Paleocene to Early Eocene warming trend
- Earth system modeling broadly reproduces mid‐Paleogene warming, δ^13^C and CCD trends via increases in volcanic CO_2_ outgassing and weathering
- Earth system modeling suggests the long‐term position of the CCD is decoupled from global marine carbonate burial and weathering inputs

## 1 Introduction

On million year timescales, the partial pressure of atmospheric carbon dioxide (*p*CO_2_) and global temperature is widely assumed to be regulated primarily by global rates of silicate mineral weathering. If this were not the case, even relatively small changes in volcanic outgassing would give rise to repeated and unbounded approximately million year timescale swings in *p*CO_2_ and climate (Berner et al., 1983; Berner & Caldeira, 1997; Kump et al., 2000; Walker et al., 1981). These are not observed in the Cretaceous‐Recent geological record, for which we have relatively continuous and high‐resolution deep‐sea climate records. Yet silicate weathering is just one part of the system stabilizing our long‐term climate. The production, preservation, and burial of organic carbon (Kump & Arthur, 1999) and/or low‐temperature crustal alteration (Alt & Teagle, 1999) may exert additional important long‐term negative feedbacks on Earth's climate. In addition, direct evidence for the link between *p*CO_2_, climate, and silicate weathering rates (as estimated by variations in strontium (Palmer & Elderfield, 1985) and lithium isotopes (Misra & Froelich, 2012) in marine carbonates) is currently ambiguous, with multiple possible interpretations of weathering proxy data sets. For example, Large Igneous Province activity may dampen any potential weathering signal in Sr isotope records (Hodell et al., 2007), and flat Li isotope signals have been variably interpreted as tectonic limitation of weathering (Froelich & Misra, 2014; Misra & Froelich, 2012) or changes in the global denudation regime (Li & West, 2014). Thus, constraining the role of silicate weathering is central to a full understanding of Earth's climate history.

A conceptually simple approach to constraining global weathering is to consider the mass balance of the carbonate system and to utilize sedimentary indicators of changes in this balance, such as the carbonate compensation depth (CCD). The basis for understanding the CCD rests in the fact that the stability of calcium carbonate (CaCO_3_) decreases with increasing pressure (i.e., depth). Carbonate secreted by plankton near the ocean surface and transported to the seafloor will begin to dissolve once it exceeds the depth of the calcite saturation horizon (CSH)—the physical‐geochemical horizon of neutral CaCO_3_ stability. Somewhere below this depth, the sedimentary CaCO_3_ dissolution flux from the sediments will equal the supply of new CaCO_3_. This depth is termed the CCD (Farrell & Prell, 1991; Ridgwell & Zeebe, 2005; Zeebe & Wolf‐Gladrow, 2001); at depths greater than this, sediments become devoid of CaCO_3_ at steady state. Pore water organic carbon respiration can also influence sedimentary carbonate dissolution and sediments at depths shallower than the CCD may show carbonate dissolution fluxes due to respiration in sediment pore waters if there is a large supply of organic matter to the seafloor (Archer, 1996). The CCD can vary between ocean basins as a function of large‐scale patterns in ocean circulation and consequent water mass aging gradients. Prior to the proliferation of planktic calcifiers in the open ocean in the Mid‐Mesozoic (Bown et al., 2004; Hönisch et al., 2012) and the consequent oversupply of biogenic carbonate to the seafloor relative to global weathering (Ridgwell, 2005; Zeebe & Westbroek, 2003) deep‐sea carbonate compensation would not have occurred and hence no CCD can be defined.

The CCD is of particular interest in understanding past changes in global carbon cycling because it is a sedimentary feature that can be reconstructed from the geological record (e.g., Farrell & Prell, 1991; Van Andel, 1975). For instance, during the geologically rapid (several kiloyears; Kirtland Turner & Ridgwell, 2016; Zeebe et al., 2016), carbon release associated with the Paleocene‐Eocene Thermal Maximum (PETM, ~56 Ma), the CCD shoaled—evidence for neutralization of emitted CO_2_ by sedimentary CaCO_3_ dissolution and a carbonate system temporarily out of equilibrium (Hönisch et al., 2012). However, on longer timescales (hundreds of thousands to millions of years), the burial of CaCO_3_ in marine sediments depends on, and indeed must generally balance, the input of the terrestrial weathering products—predominantly Ca^2+^ (and Mg^2+^) HCO_3_^−^ (Ridgwell & Zeebe, 2005; Zeebe & Westbroek, 2003). The global marine CaCO_3_ budget is also influenced by CaCO_3_ burial on continental shelves (Berger & Winterer, 1974), but greater weathering flux can be counterbalanced by increased preservation of CaCO_3_ in deep‐sea sediments. Increased deep‐sea preservation would be expected to result in a deeper CCD, providing a potential proxy for global weathering rates and ocean carbonate saturation (Kump et al., 2009). In this way, the multimillion year CCD behavior fundamentally differs from short‐term CCD responses such as the PETM, when CO_2_ release outpaced weathering feedbacks (e.g., see Hönisch et al., 2012) and global carbonate burial rates were substantially suppressed compared to weathering inputs. In contrast, on multimillion year timescales, global carbonate burial should track weathering.

**Figure 1:**
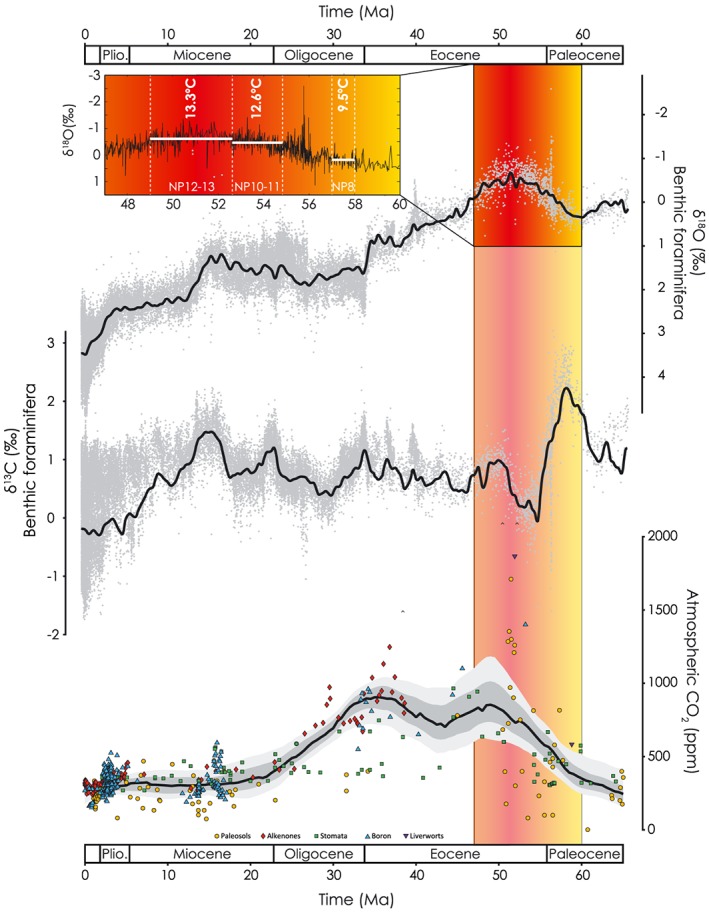
Climatic context for the LPEE. Cenozoic records of benthic foraminiferal δ^18^O (top), benthic foraminiferal δ^13^C (middle), and pCO_2_ (bottom). Isotope records are from Cramer et al. (2009). Inset shows the benthic foraminiferal oxygen isotope record across the LPEE. Three highlighted time slices are demarcated by their respective nannofossil zonations and mean temperature for each time slice is calculated via the Bemis et al. (1998) calibration. Cenozoic history of pCO_2_ is from Foster et al. (2017). Black line is most likely LOESS fit through the data; 68% and 95% confidence intervals shown as dark and light gray bands.

Its promise as a constraint on global weathering rates has led to extensive use of the position of the CCD in the reconstruction of Cenozoic changes in marine carbon cycling, ocean chemistry, and atmospheric *p*CO_2_ (e.g., Anagnostou et al., 2016; Komar et al., 2013; Roberts & Tripati, 2009; Stuecker & Zeebe, 2010; Tripati et al., 2009; Tyrrell & Zeebe, 2004). Here, we assess the interpretation of observed changes in the long‐term CCD and the strength of its link to climate change and global weathering rates, taking a multimillion year interval of progressive warming from the late Paleocene (~58 Ma) through early Eocene (~49 Ma; “LPEE”; Figure 1) as an illustration. Bottom water δ^18^O values in benthic foraminiferal carbonate across this interval decreased by nearly 0.8‰ (Cramer et al., 2009; Zachos et al., 2008), corresponding to a ~4 °C warming in the absence of any continental ice (Figure 1). This warming culminated in the Early Eocene Climatic Optimum (EECO; Zachos et al., 2001), characterized by peak Cenozoic bottom water temperatures (Cramer et al., 2009; Zachos et al., 2001, 2008). Multiple lines of evidence suggest that EECO warmth may be related to higher *p*CO_2_ as a consequence of elevated volcanic outgassing and/or a reduction in net organic carbon burial (Komar et al., 2013). Estimates of *p*CO_2_ roughly double (Beerling & Royer, 2011) between ~55 and ~49 Ma, and the δ^13^C values of benthic foraminifera trend toward more depleted values from ~58–53 Ma (Cramer et al., 2009; Figure 1). Concurrent with the early Eocene temperature rise was a trend toward depleted ^87^Sr/^86^Sr, which has been attributed to emplacement of the North Atlantic Igneous Province (Hodell et al., 2007)—a potential driver of increased volcanic outgassing rates (Eldholm & Thomas, 1993).

**Figure 2:**
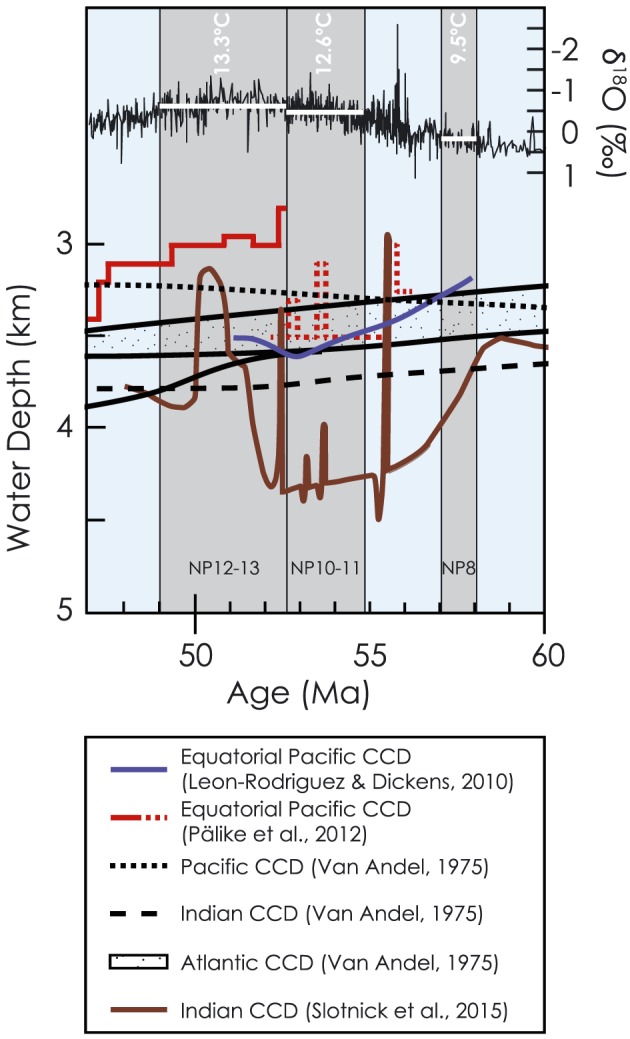
Previous CCD reconstructions across the LPEE. Black lines are from an early global compilation by Van Andel (1975), red line from the Pacific equatorial age transect of Pälike et al., 2012, blue line from the equatorial Pacific record of Leon‐Rodriguez and Dickens (2010), and brown line from the Indian Ocean record of Slotnick et al. (2015). CCD = carbonate compensation depth.

High global surface temperatures should have driven an increase in the rate of weathering, which would generally be expected to result in a deeper CCD. However, the only previous global reconstruction of CCD changes during the Paleocene and Eocene by Van Andel (1975; Figure 2) predated four decades of intense ocean drilling and thus relied on sparse data. Since this seminal paper, the position of the CCD across the LPEE has been reassessed regionally, based on clusters of sites (Hancock et al., 2007; Leon‐Rodriguez & Dickens, 2010) or depth transects (Pälike et al., 2012; Figure 2). These regional studies show discrepancies in the absolute position of the CCD. Specifically, the two studies based on different Equatorial Pacific sites disagree by over 500 m across the EECO (from ~53–51 Ma; Leon‐Rodriguez & Dickens, 2010; Pälike et al., 2012). These regional studies generally agree that the CCD was slightly shallower in the late Paleocene (~57 Ma) than at the onset of the EECO (55–53 Ma), but the trends diverge markedly, entering peak EECO warmth (52–50 Ma; Figure 2).

There are significant challenges in extrapolating from basin‐specific CCD reconstructions to a global CCD record. Changes in global ocean circulation patterns can drive regional changes in the CCD, even with little change globally. For example, the deep‐sea CaCO_3_ burial pattern from the Last Glacial Maximum to the present day (Catubig et al., 1998) shows profound CCD changes in the Atlantic, associated with local changes in ocean circulation (Chikamoto et al., 2008), yet no change in global burial rates between glacial and interglacial (Anderson & Archer, 2002). Circulation changes may also have regionally amplified the dramatic PETM CCD shoaling at Walvis Ridge in the South Atlantic via concurrent reductions in local deepwater formation (Zeebe & Zachos, 2007). In addition, changes in a site's latitude due to plate motion may correlate with differences in overlying productivity, biasing the CCD interpretation (Lyle, 2003).

**Figure 3:**
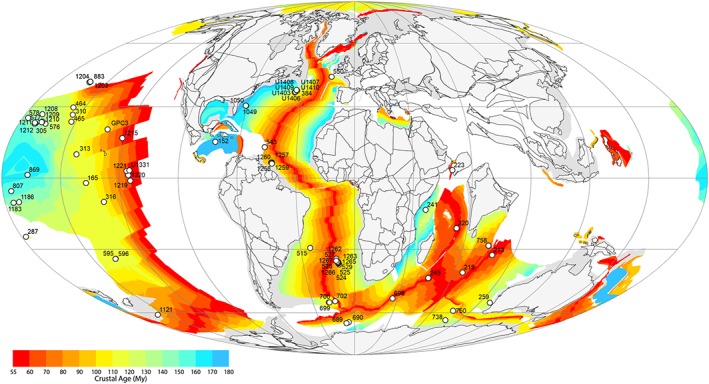
Paleogeography and deep‐sea drilling coverage. Paleogeography after Scotese (2010) showing locations of deep‐sea drilling sites, which appear in at least one of the three late Paleocene to early Eocene time slices presented.

Only an appropriately (area) weighted average CCD can reflect changes in global weathering rates. One could infer from (a) the dominance of the Pacific Ocean in the Paleogene in terms of area (Figure 3) and (b) existing estimates of either slightly deeper (Leon‐Rodriguez & Dickens, 2010) or slightly shallower (Pälike et al., 2012) Pacific CCD in the early Eocene than in the late Paleocene that relatively little CCD movement occurred across the LPEE globally (Figure 2). However, global carbon cycle box models predict a CCD deepening of >1 km (Komar et al., 2013) to explain observed long‐term warming and δ^13^C trends in terms of greater CO_2_ outgassing due to enhanced volcanism and consequently higher weathering rates. Such a large deepening is seen only in the northeastern Indian Ocean (Figure 2; Slotnick et al., 2015), for the interval 58 through 52 Ma. Here we assess the impact of LPEE climate change on the CCD by (a) reconstructing the CCD using time slices of deep‐sea sedimentary wt% CaCO_3_ and (b) modeling the position of the CCD in response to progressively increasing atmospheric CO_2_ with the Earth system model cGENIE.

## 2 Methods

### 2.1 Data Compilation

We compiled published wt% CaCO_3_ measurements and reconstructed paleodepths for Deep Sea Drilling Project (DSDP), Ocean Drilling Program (ODP), and Integrated Ocean Drilling Program (IODP) sites spanning the late Paleocene to early Eocene (Tables S1–S3 in the supporting infomation). Sites with upper Paleocene or lower Eocene sediments were included in our data set using the following criteria: (1) paleodepths deeper than 1,000 m, (2) no indication of reworking, and (3) existing wt% CaCO_3_ measurements. Our compilation thus includes 75 DSDP, ODP, and IODP sites. The data set includes three time slices: (1) the uppermost Paleocene (corresponding to nannofossil biozones NP7/8 or roughly coeval foraminiferal biozone P4c), (2) the lowermost Eocene (zones NP10/11 or foraminiferal zone P6a), and (3) the interval spanning peak warmth of the EECO (NP12/13 and if needed foraminiferal zone P7/P8). (The latter time slice is shown in the supporting information [SI] figures only). Zone NP9 (P5) hosts the PETM, which was deliberately excluded. Reported wt% CaCO_3_ values are averages of all measured values within a given time slice for each site. Biostratigraphy and wt% CaCO_3_ values are derived from DSDP/ODP/IODP volumes, primarily downloaded via the Janus web database (http://www‐odp.tamu.edu/database/) and the Ocean Drilling Stratigraphic Network database (http://www.odsn.de/) and supplemented by Hancock et al. (2007) for Site 259. For deep‐sea clay sites without wt% CaCO_3_ measurements but lacking CaCO_3_ in sedimentological descriptions, 0 wt% was assumed. Paleolatitude and paleolongitude for each site were computed for 55 Ma using the “Point Tracker (v. 7) for Windows” software package (www.scotese.com).

Paleodepths were derived via backtracking, which accounts for thermal subsidence and sediment unloading based on empirical approximations (from Stein & Stein, 1992), but ignoring possible effects due to dynamic topography (Campbell et al., 2018). We employ an empirically fitted curve with a free initial ridge height following Cramer et al. (2009), but using a simplified sediment unloading term that assumes linear sedimentation rates (see SI). Inputs are basement age, current water depth, and total sediment thickness. Basement ages are taken from Müller et al. (2008), with the exception of ODP Sites 689 and 690, for which a basement age of 83 Ma is taken from Barker et al. (1988). Present‐day water depths and sediment thicknesses are taken from the DSDP/ODP/IODP Initial Reports. For sites where basement was not identified or unclear from seismic data, the total drilled sediment thickness was taken as a minimum estimate. See SI for discussion of paleodepth error.

**Figure 4:**
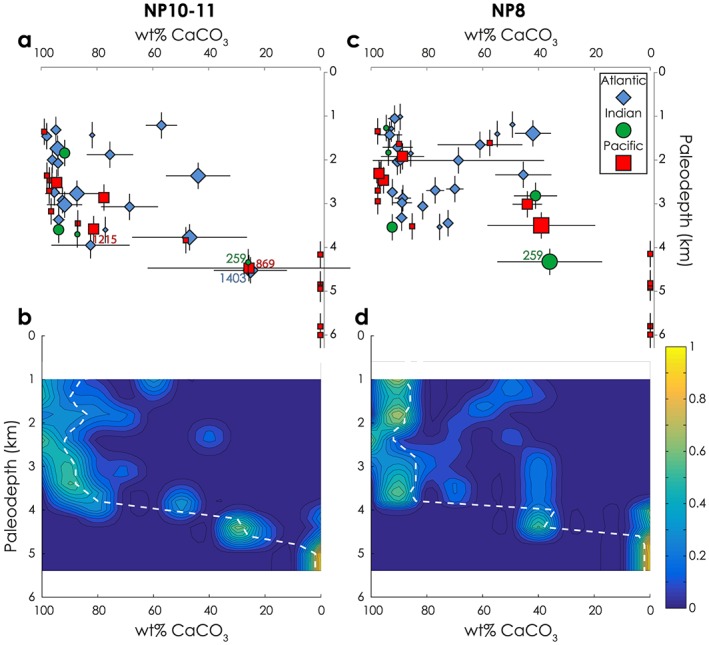
Reconstructed carbonate compensation depth across the LPEE. (a, c) Paleodepth versus wt% CaCO_3_ for two time slices spanning the LPEE (NP8 and NP10‐11). Symbol size denotes the number of wt% CaCO_3_ measurements averaged for each site (small: 1 measurement, medium: 2‐9 measurements, large: ≥10 measurements). Horizontal error bars represent 1σ from the mean wt% CaCO_3_ recorded at each site within the time slice. Vertical error bars represent ±300‐m paleodepth (an estimate derived by trebling the paleodepth error estimate for sites <25 Ma (Sclater et al., 1985) or doubling the ±150 m suggested by Van Andel, 1975 and Van Andel et al., 1975 for sites underlain by crust older than a few million years at the time of paleodepth reconstruction). (b, d) Same data set as above, but contoured and normalized to highlight where the data at any given depth fall in wt% CaCO_3_ space (see section 2.1). Color represents “density” of normalized weight and sums to 1 at any given depth. The white dashed lines denote the median weight percent value with 50% of the data at higher or lower weight percent values for any given depth.

Contour plots of wt% CaCO_3_ against paleodepth assist in visualizing the CCD for each LPEE time slice. These plots (Figures 4 and 6) highlight the depth interval over which the preponderance of sites transition from high to low wt% CaCO_3_. We binned raw wt% CaCO_3_ into depth windows of 1,000 m, starting at 1,000 m, and moving deeper in increments of 100 m. For each depth window, the CaCO_3_ wt% bins span intervals of 10 wt%. Within each depth bin, wt% CaCO_3_ values are weighted using a triangular weighting function that assigns full weight to data at the center of the depth bin, with weight declining linearly to zero for data at the edges of the depth bin. The depth bin is then normalized to 1 and contoured, highlighting where the majority of the data within a depth falls in wt% CaCO_3_ space. We also overlay a vertical dotted line that delineates the median weight percent at any given depth, which indicates the depth over which our data transition from predominantly carbonate replete to predominantly carbonate poor.

### 2.2 Statistics

Having compiled wt% CaCO_3_ against paleodepth for each of the LPEE time slices, we next tested whether the position of the CCD differed between each time slice by determining whether wt% CaCO_3_ varied with paleodepth through time. To do this, we implemented a suite of Generalized Linear Mixed Models (GLMM) using the glmmAMDB package (version 7.2.12; Skaug et al., 2014) in Rstudio (version 0.97.336). Linear Mixed Models use regression analysis to parse how variation in a response, or dependent, variable relates to multiple explanatory, or independent variables, as well as random terms concurrently; that is, these statistical analyses take into account the potential effects of multiple factors on a particular response at the same time. GLMMs are an extension of linear mixed models that allow data to be modeled according to non‐normal distribution functions. In all models, each individual wt% CaCO_3_ measurement was entered as the response variable. Therefore, to account for sites with multiple samples within a time slice, we always included “site” as a random variable. Our percentage data most closely resembled the binomial distribution function; that is, this function was the best “fit” for our data, which we therefore applied in all models. In the first model, we included all paleodepths, and interacted paleodepth (continuous variable) with time slice (three categories: one, two, and three). Given that previous work (Hancock et al., 2007; Penman et al., 2016) and our data compilation indicated that Sites 259, 869, and 1403 were particularly significant because of paleodepths very close to the modern CCD, we reran this full model three additional times, removing one site from each model iteration. We additionally ran a similar suite of models, including only paleodepths below (a) 3,000 and (b) 3,500 m based on the assumption that data from these deeper sites would be particularly significant for constraining the CCD. In these models, time slice was entered as the only explanatory variable, but all other parameters were identical to above. We again removed each of the three sites (259, 869, and 1403) from these models to determine if the results were driven by site biases.

### 2.3 Modeling

#### 2.3.1 Model Configuration

We utilize “cGENIE”—an Earth system model of intermediate complexity comprising: a 3‐D dynamic ocean circulation model with simplified “energy and moisture” balance atmosphere (and sea ice; Edwards & Marsh, 2005), a representation of the biogeochemical cycling of major nutrients, trace elements, and isotopes in the ocean (Ridgwell, 2007), plus components accounting for interactions between ocean and marine sediments (Ridgwell & Hargreaves, 2007) and terrestrial weathering (Colbourn et al., 2013), and hence closing the geological cycle of carbon.

cGENIE includes a full representation of the marine carbon cycle, which shapes water column carbonate saturation state (Ω_carb_) and also includes a representation of marine sedimentary carbon cycling, namely, sedimentary aerobic respiration of organic carbon and CaCO_3_ dissolution (Ridgwell, 2007), which can influence pore water Ω_carb_. The details of the ocean and sediment carbon cycle processes have been described elsewhere (e.g., Kirtland Turner & Ridgwell, 2013; Ridgwell, 2007; Ridgwell & Hargreaves, 2007; Ridgwell, Hargreaves, et al., 2007; Ridgwell, Zondervan, et al., 2007), so we focus on the specific configuration of the terrestrial rock weathering model. Here, the global fluxes of alkalinity (ALK) and dissolved inorganic carbon (DIC) resulting from carbonate and silicate rock weathering (plus associated Ca^2+^ and carbon isotopes) are estimated and routed to the coastal ocean in a pattern based on modern watersheds (Edwards & Marsh, 2005). In this study, we select parameterizations for these fluxes determined solely by, and in feedback with, mean global land surface temperature as in Colbourn et al. (2013). In this, temperature dependence of CaCO_3_ weathering follows Berner (1994; derived by correlating temperatures and bicarbonate concentrations of groundwater), whereas the relationship between silicate weathering and temperature is based on laboratory studies of the impact of temperature on the dissolution of Ca and Mg silicates (Brady, 1991). Note that we chose a zeroth order description (temperature dependence only) and omit potential additional modifiers accounting for the influence of, for example, runoff (Berner et al., 1983) or terrestrial productivity (Berner, 1991). Despite this, the specific combination of weathering feedback and marine sediment burial used gives rise to a millennial‐scale CO_2_ response comparable to that produced by other Earth system models (Archer et al., 2009). The dynamics and climate feedback sensitivity in cGENIE of these and alternative possible weathering parameterizations are described and assessed in detail by Colbourn et al. (2013, 2015).

We configure cGENIE using a late Paleocene/early Eocene bathymetry, continental configuration, and carbon cycle as in Kirtland Turner and Ridgwell (2016) and Gibbs et al. (2016). We use the following global carbon cycle conditions: major cations have initial mean oceanic concentrations of 18.2 mmol/kg [Ca^2+^], 29.9 mmol/kg [Mg^2+^], and 15.0 mmol/kg [SO_4_^2−^] following Panchuk et al. (2008). The effect of elevated (compared to modern) Paleogene [Ca^2+^] and lower [Mg^2+^] in influencing ocean carbonate chemistry and global carbon cycling is included by assuming that deviations of [Mg^2+^] from modern modify K_1_, K_2_, while different [Mg^2+^]/[Ca^2+^] affects K_sp_, as described in Panchuk et al. (2008). The marine biological CaCO_3_:POC export ratio is assumed globally uniform and set to a value of 0.200, a value determined to give rise to an optimal fit to global distributions of upper Paleocene wt% CaCO_3_ by Panchuk et al. (2008). Finally, the air‐sea gas transfer coefficient is increased from 0.31 to 0.52 to rescale (CO_2_) gas transfer to the inferred modern value of ~0.058 mol·m^−2^·year^−1^·μatm^−1^.

The model was initialized with a value of alkalinity (1,975 μmol·eq·kg^−1^) chosen to produce a mean sediment CaCO_3_ content (below 176‐m water depth) of ~47 wt% and a global deep‐sea sediment CaCO_3_ burial rate of 14.7 Tmol CaCO_3_/year to closely match the optimal upper Paleocene sediment distribution determined by Panchuk et al. (2008). Note that the calculation of fractional calcium carbonate preservation (Archer, 1991; Ridgwell, 2007) only allows for organic carbon to be respired aerobically and, moreover, requires that organic carbon reaching the seafloor is completely respired within the sediments (Archer, 1991). Note also that to provide consistency with the study of Panchuk et al. (2008), when necessary, we modified the seafloor depth (pressure) used to calculate carbonate stability in the sediment model at the specific model‐equivalent ocean drilling data locations identified. In an initial spin‐up phase, as described in Ridgwell and Hargreaves (2007), the ocean‐atmosphere carbon cycle is forced “closed” with global weathering fluxes tracking sedimentary burial of CaCO_3_ at all times and no bioturbation in the surface sediment layers. After 20 kyr of this closed system spin‐up with atmospheric CO_2_ kept at 834 ppm (~×3 preindustrial *p*CO_2_) and its δ^13^C at −4.9‰, DIC is constrained at 1,977 μmol/kg. The resulting equilibrium climatology is summarized in Ridgwell and Schmidt (2010). In a second follow‐on phase of spin‐up, the model was run for 200 kyr as an “open” system with temperature‐dependent silicate and carbonate weathering enabled (Archer et al., 2009; Colbourn et al., 2013) and atmospheric CO_2_ and δ^13^C free to evolve. The initial global Ca^2+^ weathering flux was diagnosed from the burial flux (14.7 Tmol Ca^2+^/year) at the end of the first spin‐up phase and split equally between silicate (7.35 Tmol Ca^2+^/year) and carbonate (7.35 Tmol Ca^2+^/year) weathering. An initial flux of volcanic CO_2_ of 7.35 Tmol C/year (δ^13^C of −6.0‰) was specified to balance consumption by silicate weathering. Bioturbation and hence mixing of the surface sediments was then enabled (following the procedure of Ridgwell & Hargreaves, 2007). The δ^13^C signature of carbonate weathering was set to balance the long‐term ^13^C budget, requiring an enriched signature of 13.58‰ because the model does not include organic carbon burial. Following the spin‐up to balance the geologic carbon cycle, a subsequent control experiment showed drift in atmospheric CO_2_ of less than 2 ppm over 2 Myr.

#### 2.3.2 Extraction of Modeled CCD and CSH

For determining the simulated carbonate compensation depth in the cGENIE model, we adopted the methodology of Goodwin and Ridgwell (2010). Rather than identifying the model depth at which the sedimentary carbonate deposition and dissolution fluxes are equal, we instead assume that the CCD is coincident with the 20 wt% CaCO_3_ isoline following Van Andel (1975). (This alternative definition of the CCD is necessary for paleo‐applications as there is no possible way to reconstruct carbonate deposition and dissolution fluxes from a sediment core.) However, the chances of finding a location with almost exactly 20 wt% CaCO_3_ on a discretized grid of less than 1,000 ocean grid points (e.g., Figures S5 and S6) is relatively small, and even if one or a few such points existed, there would be a substantial risk that they would not be representative of the global CCD. We hence interpolated in depth to the 20 wt% boundary using the much larger number of pairs of locations that exhibit carbonate content either side of this value (i.e., one lower and one higher than 20 wt%). For grid points judged to lie below the CCD (i.e., >20 wt% CaCO_3_), immediately adjacent cells (including diagonal relationships) are tested as to whether they lie above the CCD (i.e., >20 wt% CaCO_3_). Grid points bordering land (i.e., continental slope) are excluded so as to avoid biasing by relatively few, but highly productive/upwelling continental margin locations. We also exclude pairs of adjacent grid points that are separated in depth by more than 2000 m to avoid excessive interpolation, but find that our results are virtually independent of the value of this cutoff. Setting a maximum depth separation of 1,000 versus 6,000 m leads to essentially the same answer for the global mean CCD in the model. Finally, CCD‐spanning pairs with the below‐CCD point having <1 wt% are excluded, together with pairs characterized by an above‐CCD point having >50 wt%. The purpose of imposing a maximum wt% CaCO_3_ in the above‐CCD point is to avoid distortions arising from averaging across the lysocline, above which the change in wt% CaCO_3_ with decreasing depth is much less steep. For each pair of CCD‐spanning adjacent model grid points, we then calculate the CCD depth as an average weighted by how close the carbonate content of the cell is to the 20 wt% boundary. In other words, in a grid point pair characterized by one point with 19 wt% CaCO_3_ and one with 50 wt%, the CCD depth will be much closer to the depth of the 19 wt% CaCO_3_ location. A typical distribution of CCD‐spanning grid point pairs is shown in Figure S6. The final estimate of the global mean CCD in the model is then simply the arithmetic mean of the interpolated CCD separating each grid point pair. For determining the depth of the carbonate saturation horizon (CSH) in the model, a quadratic regression is fit to the global distribution of Δ[CO_3_^2−^] (the carbonate ion concentration relative to local saturation) versus depth in the model for depths greater than 1,000 m, and the intersection of this line with Δ[CO_3_^2−^] = 0 (saturation) calculated.

#### 2.3.3 Experimental Setup

Using the above configuration, we ran five ensembles of experiments designed to test controls on the position of the CCD in cGENIE. Ensemble 1 consists of a series of experiments with volcanic outgassing rate modified by factors ranging from 0.6 to 2.0 and run to a new steady state (2 Myr) as an open system. Ensemble 2 isolates the roles of [DIC] and [ALK] on the CCD by fixing radiative forcing so that global temperature is the same in each experiment and hence not a function of atmospheric CO_2_. Ensemble 2 was created by restarting each experiment from ensemble 1 and running for 20 kyr as a “closed system” with no further gains to or losses from the ocean‐atmosphere (Ridgwell & Hargreaves, 2007), thus preserving the [DIC] and [ALK] of the respective ensemble 1 experiments. We use radiative forcing of 3 times preindustrial (equivalent of 834 ppm) for all experiments, equivalent to our open‐system spin‐up. Fixing radiative forcing also eliminates differences in ocean overturning circulation between each experiment. In contrast, ensemble 3 isolates the role of temperature and ocean circulation on the CCD. All experiments in ensemble 3 were restarted from the 3 times preindustrial CO_2_ experiment from ensemble 1 with radiative forcing set to match the equivalent atmospheric CO_2_ from each ensemble 1 experiment. Each experiment was run for 20 kyr as a closed system so that [DIC] and [ALK] were relatively invariant. Ensemble 4 tests the impact of fixed global weathering rates on the CCD. All experiments in ensemble 4 were restarted from the 3 times preindustrial CO_2_ experiment from ensemble 1, but with atmospheric CO_2_ fixed equal to the CO_2_ value reached at the end of each equivalent ensemble #1 experiment. Ensemble 4 experiments were run for 20 kyr.

Finally, to test the potential influence of grid resolution on our modeled CCD, we ran one final ensemble (ensemble 5), duplicating ensemble 1 but replacing the bathymetry grid used for the calculation of pressure and carbonate stability in the sediment model (see Ridgwell, Hargreaves, et al., 2007, for an example). Compared to the standard sediment model grid derived by discretizing the underlying coupled GCM bathymetry (Tindall et al., 2010) according to the 16 depth layers of the ocean circulation model as used in Ridgwell and Schmidt (2010), we instead discretized at 64 levels and with a maximum 6,000 m (rather than 5,000 m) ocean depth. The alternative sediment model depth grid (Figure S8) is considerably smoother than the original (Figure S7).

## 3 Results

### 3.1 Reconstruction of the LPEE CCD

While we reconstructed three multi‐million year CCD time slices corresponding to nannofossil zones NP8, NP10‐11, and NP12‐13, most of the change in deep ocean temperature (as indicated by benthic foraminifera δ^18^O) occurs between the NP8 and NP10‐11 time slices, and these two time slices also show a larger difference in δ^13^C than comparing NP8 to NP12‐13. Hence, we focus most of our comparison and discussion on the NP8 versus NP10‐11 time slices, though results for NP12‐13 are provided in SI.

Paleodepth versus wt% CaCO_3_ for the NP8 and NP10‐11 time slices are plotted in Figures 4a and 4c (for NP12‐13, see Figure S1). Figures 4b and 4d show the same data in normalized contour plots. (The color shows the density of underlying data and sums to 1 across any given depth.) The dashed white lines indicate that a switch from a majority of high wt% sites to a majority of low weight percent sites occurs between ~4,000 and 4,500 m for both time slices. We hence interpret ~4,000 m as the upper limit for the global mean paleo‐CCD. We have confidence that the CCD was not shallower than 4,000m, as there are numerous records from shallower depths replete with carbonate. Below 4,000 m the sediments are predominantly carbonate‐free, but it is important to note that the data are sparser and almost entirely restricted to the Pacific basin. For these reasons, we have comparatively lower confidence about the maximum possible depth of the global mean CCD but consider it unlikely that it was much deeper than 4,000m in the Pacific basin. The most notable difference between our two time slices among deep sites occurs at Atlantic IODP Site U1403, where CaCO_3_ burial commenced in the earliest Eocene (NP10‐11 time slice; Expedition 342 Scientists, 2012; Figures 2 and 4). The temporal evolution of wt% CaCO_3_ within each time slice at each site is plotted in Figure S2. Importantly, there are no consistent trends toward increasing or decreasing wt% CaCO_3_ within any of our time slices.

Our statistical models indicate that time slice does not significantly correlate to wt% CaCO_3_. Specifically, when we included data from all paleodepths, there was no significant interaction between time slice and paleodepth (GLMM: *p* > 0.2 for all models), nor was there any overall effect of time slice (GLMM: *p* > 0.2 for all models). The interactions between paleodepth and time slice were identical or even more insignificant when any of the three deepest carbonate bearing sites (259, 869, or U1403) were removed (GLMM: *p* > 0.2; GLMM: *p* > 0.2; GLMM: *p* = 1.0, respectively). Likewise, when considering just data with paleodepths below 3,000 m, there was no detectable effect of time slice (GLMM: *p* > 0.9), nor was there when we removed Site 259 (GLMM: *p* > 0.9), Site 869 (GLMM: *p* > 0.9), Site U1403 (GLMM: *p* = 1.0), or all three sites together (GLMM: *p* = 1.0). The models revealed almost identical results when we considered data with paleodepths below 3,500 m. Thus, the comparatively high abundance of sites at shallower paleodepths does not mask statistical differences in the CCD between time slices. In sum, the three time slices are statistically indistinguishable.

Though statistically indistinguishable, visual comparison of the median wt% CaCO_3_ for any given paleodepth between the time slices (white dashed lines, Figure 4) shows they are not identical. The transition interval from a high median wt% CaCO_3_ to a low median wt% CaCO_3_ occurs at a slightly shallower paleodepth in NP8 than in NP10‐11. The transition to a majority of sites with carbonate content below 20 wt% occurs 160 m deeper for NP10‐11 than for NP8. However, we note that this degree of deepening is likely within the error of paleodepth estimates (see discussion in SI). Our results are thus generally consistent with a CCD deepening of a few hundred meters between 58 and 49 Ma in the equatorial Pacific as suggested by Leon‐Rodriguez and Dickens (2010). Our global‐scale analysis confirms that the CCD deepened, at most, very modestly during the LPEE. However, this modest deepening contrasts with the >1‐km CCD deepening suggested previously by models (Komar et al., 2013) seeking to explain LPEE climatic trends as a result of enhanced volcanism.

### 3.2 Interpreting the CCD as a Global Carbon Cycle Proxy

What explains the three main lines of marine geological observations across the LPEE: minor CCD deepening, global warming, and δ^13^C decline? Our CCD compilation lends itself to several possible interpretations: (1) The CCD was relatively unresponsive to increased weathering across the LPEE (Kato et al., 2011; Peucker‐Ehrenbrink & Ravizza, 2000); (2) the CCD is directly coupled to global weathering rates and the relatively minor difference in the CCD across time slices reflects only a minor increase in global weathering rates, despite the observed rise in temperatures (Froelich & Misra, 2014), or (3) increased weathering across the LPEE was accommodated by a commensurate increase in shelf CaCO_3_ burial, resulting in a relatively stable CCD. For the purposes of this study, we set aside one further possibility—(4) that the CCD deepened considerably (especially outside of the Pacific), but we missed this because of sparse data coverage below ~4,000 m in other ocean basins (Figure 4). This possibility can only be addressed by further drilling of open ocean sites at greater paleodepths. However, our data compilation excludes the possibility of kilometer‐scale deepening of the CCD in much of the Pacific. Given the areal dominance of the Pacific basin in the early Paleogene (Figure 3), this, in turn, excludes the possibility of a global mean kilometer‐scale CCD deepening.

**Figure 5:**
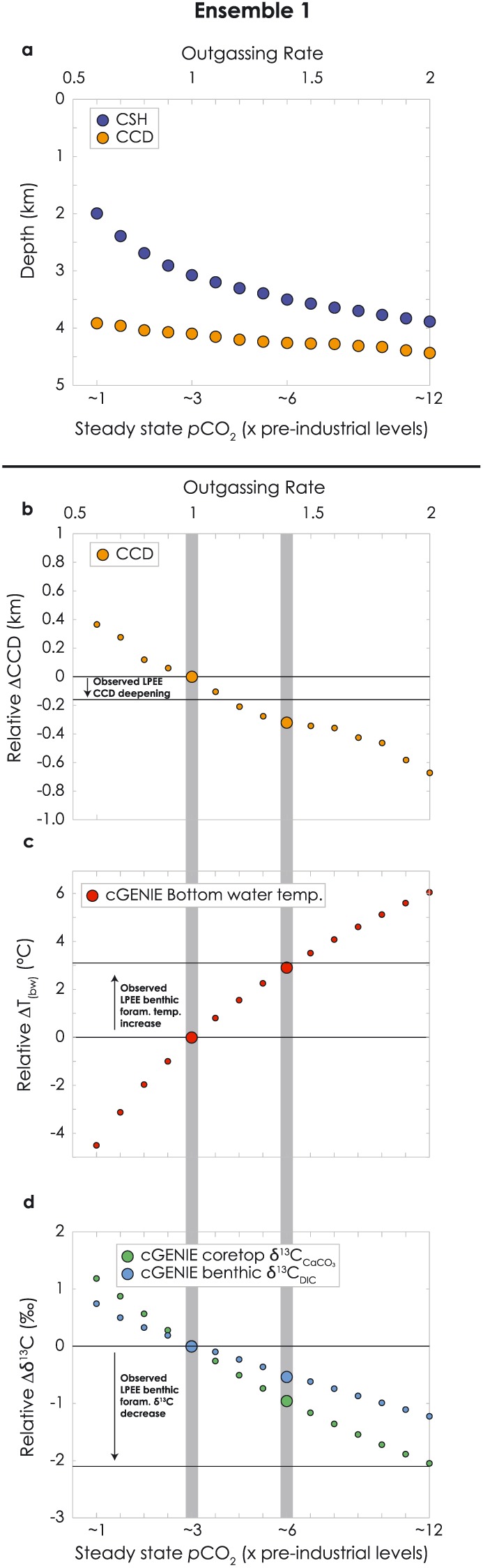
Relationship between CCD, CSH, bottom water temperature, δ^13^C, and climate in cGENIE relative to the “baseline” experiment at 3 times preindustrial pCO_2_. (a) Depth of CCD and CSH extracted from ensemble 1 experiments at various outgassing rates/atmospheric pCO_2_ levels; (b–d) CCD depth, mean bottom water temperatures, and mean core top δ^13^C_CaCO3_ from ensemble 1 experiments. Vertical gray bars highlight the best fit experiments for the pre‐EECO (NP8) bottom water temperatures (left) and EECO (NP10‐11) bottom water temperatures (right). CCD = carbonate compensation depth; CSH = calcite saturation horizon; EECO = Early Eocene Climatic Optimum.

Possibility (1), that progressive increases in atmospheric *p*CO_2_, surface temperature, and global weathering rates failed to result in any substantial deepening of the CCD, was first suggested by Pälike et al. (2012). Our results from ensemble 1 test this hypothesis by evaluating the position of the CCD as a function of changes in volcanic outgassing when including a temperature‐dependent weathering feedback (Tables S4–S18). From each experiment, we extract the CSH and CCD (Figure 5) following Goodwin and Ridgwell (2010). Our results are consistent with Pälike et al. (2012), in that the CCD changes little despite large changes in atmospheric CO_2_ and global temperature. Compared to the CCD in the ×3 *p*CO_2_ experiment, the CCD in ~×12 *p*CO_2_ experiment is just 300 m deeper. The change in deep ocean temperature of 3.1 °C between NP8 and NP10‐11 is best represented by comparing the ×3 *p*CO_2_ and ~×6 preindustrial *p*CO_2_ experiments. In the model, the CCD is 160 m deeper between these two experiments, consistent with our data compilation. The CSH, in contrast, is much more sensitive to changes in *p*CO_2_ and climate (Figure 5); at ~×6 preindustrial *p*CO_2_ the CSH is >425 m deeper than at ×3 *p*CO_2_.

**Figure 6:**
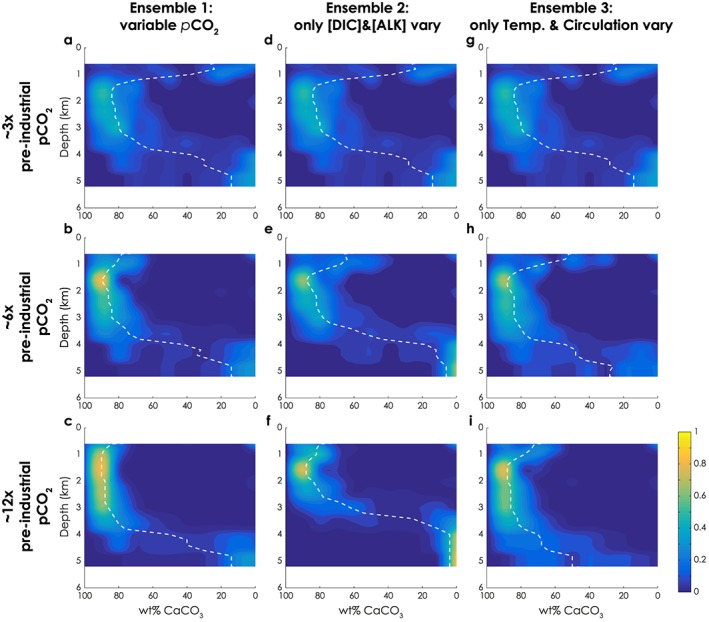
Competing climate influences on carbonate burial in cGENIE. Weight percent carbonate and depth data from model output contoured in the same manner as paleodata in Figure 1 (see section 2.1). (a–c) Three steady‐state experiments from ensemble 1 spanning a range of pCO_2_; (d–f) Three experiments from ensemble 2 with fixed temperature and circulation, but with variable total carbon inventory as in ensemble 1. (g–i) Three experiments from ensemble 3 with fixed total carbon inventory, but varying temperature and circulation as in ensemble 1. DIC = dissolved inorganic carbon.

These modest CCD changes occur despite the fact that we include temperature‐dependent weathering and simulate large increases in global temperatures. It is rather counterintuitive that a substantive increase in weathering rates and solute supply to the ocean should result in relatively little CCD deepening, because (1) any increase in weathering requires that additional carbonate burial occurs in order to eventually balance the system (Ridgwell & Zeebe, 2005) and (2) we ran all experiments in ensemble 1 to steady state with respect to the carbonate system. Closer inspection, however, reveals that our model accommodates the excess CaCO_3_ burial predominantly at depths above the CCD, rather than requiring the CCD to deepen. An increasingly bimodal distribution of wt% CaCO_3_ with depth develops at higher steady‐state atmospheric *p*CO_2_ (Figure 6a vs. 6b and 6c), with a sharp transition between high wt% and low wt% CaCO_3_ at around 4,000‐m water depth indicated by the white dashed lines. A similar pattern is perhaps detectable in the equivalent contour plots of our LPEE wt% CaCO_3_ data—particularly comparing NP8 to NP12‐13 (Figure S1)—although the data at these crucial depths are sparse.

**Figure 7:**
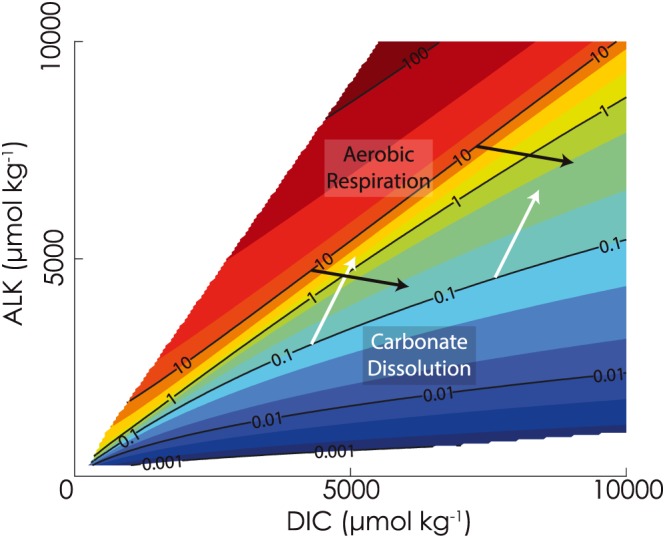
Pore water saturation state evolution in a carbon‐poor versus a carbon‐replete ocean. Lines of equal saturation state (Ω) of CaCO_3_ are plotted as a function of [ALK] and [DIC]. Two sets of vectors of equal length represent the trajectory of carbonate dissolution (white) and aerobic respiration of organic carbon (black) in saturation state space. The set of vectors that initiate at low [DIC] and [ALK] (left: a “carbon‐poor” ocean) depict larger changes in saturation state from arrow tail to arrow tip compared with the set of vectors that initiate at high [DIC] and [ALK] (right: a “carbon‐replete”) ocean. DIC = dissolved inorganic carbon.

The bimodal wt% CaCO_3_ pattern in the model occurs because sedimentary CaCO_3_ preservation is influenced by both the carbonate chemistry of the overlying ocean (Ridgwell, 2007; e.g., Figure S3) and geochemical reactions in pore waters. The dominant pore water reactions in a typical open ocean setting (and the only two pore water reactions in the cGENIE model) are aerobic respiration of organic carbon and CaCO_3_ dissolution (Archer, 1991; Ridgwell, 2007). Aerobic respiration decreases the pore water carbonate saturation state (Ω_carb_) and reduces preservation of CaCO_3_; CaCO_3_ dissolution increases the pore water Ω_carb_, buffering against further dissolution (Zeebe & Wolf‐Gladrow, 2001; Figure 7). Both reactions can occur in sediments at any water depth, but the relative importance of each reaction is depth dependent. At shallower depths (at or above the CSH) bottom waters are supersaturated (Ω_carb_ > 1), that is, not conducive to CaCO_3_ dissolution. However, at shallow depths there is still a notable flux of particulate organic matter to the sediments. (This flux decays exponentially with water depth; Ridgwell, Hargreaves, et al., 2007.) Combined with the requirement that all organic matter be remineralized aerobically, this means that aerobic respiration is the dominant reaction at depths shallower than ~2,000 m, driving pore water Ω_carb_ lower. Conversely, at greater depths below the CSH, CaCO_3_ dissolution in pore waters neutralizes the undersaturation inherited from bottom waters, elevating pore water Ω_carb_ until either (a) all carbonate is dissolved or (b) Ω_carb_ reaches 1, which inhibits further dissolution.

However, the effects of respiration and carbonate dissolution on pore water Ω_carb_ differ as a function of the whole ocean concentrations of [DIC] and alkalinity [ALK] (illustrated in Figure 7). [DIC] and [ALK] are largely controlled by weathering rates. At higher steady‐state *p*CO_2_, thus higher weathering rates (leading to higher steady state [DIC] and [ALK]), aerobic respiration causes a smaller decrease in pore water Ω_carb_ in the model, driving less dissolution at shallower water depths. Thus, more CaCO_3_ burial occurs at depths above the CSH in a warmer world with higher weathering rates. Below the CSH, higher [DIC] and [ALK] mean that neutralizing undersaturated bottom waters requires more CaCO_3_ dissolution, reducing CaCO_3_ burial. The sum of these effects is to drive the CCD shallower in a warmer world with higher weathering rates. We illustrate these processes with experiments from ensemble 2 (listed in Table S20), in which we fixed global temperature and allowed only *p*CO_2_ [DIC] and [ALK] to vary as a function of changes in volcanic outgassing (Figures 6d–6f). In this ensemble the CCD lies shallower in the experiments with higher *p*CO_2_, [DIC] and [ALK].

Yet, the effect of higher ocean temperature as a consequence of higher *p*CO_2_ should work in the opposite direction, suppressing the solubility of both CaCO_3_ and CO_2_ (Zeebe & Wolf‐Gladrow, 2001), thus increasing the preservation of carbonate at depth. We illustrate this effect using experiments from ensemble 3 (listed in Table S21), in which we varied global temperature (and hence ocean circulation) but fixed the global ocean‐atmosphere carbon inventory. In these, the CCD deepens with temperature increase (Figures 6g–6i) by an amount that approximately offsets the effect shown in ensemble 2 (Figures 6d–6f). The combination of ensembles 2 and 3 thus explains the outcome from ensemble 1—higher volcanic outgassing leading to higher *p*CO_2_, warming, and enhanced weathering results in a relatively invariant CCD.

There is one additional observational constraint across the LPEE for which we can compare model and data: δ^13^C decline. Benthic foraminifera show a decrease of ~2‰ between NP8 and NP10‐11 (Cramer et al., 2009). At steady state in our model, the ~3 °C warming between NP8 and NP10‐11 (Figure 5c) requires a 1.4 times increase in CO_2_ outgassing rate (from ×3 to ~×6 preindustrial *p*CO_2_). The associated global mean change in bulk δ^13^C_CaCO3_ predicted by the model at steady state is ~−1‰ (Figure 5d and Table S19), only about half of the observed decrease (Figure S4). The changes in benthic δ^13^C_DIC_ (Table S19) are somewhat more muted (−0.5‰), because the surface‐benthic δ^13^C gradient becomes more muted in experiments with higher outgassing rates, as the ocean becomes more carbon‐replete even at stable biological pump strength. These results show that increased volcanism and associated weathering can account for the observed LPEE trends in temperature and the CCD, but only partially explain the observed change in δ^13^C (Figures 5b–5d). However, our model experiments do not include representation of either marine or terrestrial organic matter burial. Decreased organic matter burial could drive additional decrease in benthic δ^13^C and could help explain this relatively poor fit between model and data. Eocene sediments are generally characterized by reduced burial rates of organic carbon relatively to today (Olivarez Lyle & Lyle, 2006), which may be a consequence of more intense recycling of organic matter in the water column (again, not represented in our model experiments; John et al., 2014). Yet, how organic carbon burial changed across the LPEE is not constrained by the data.

The alternative interpretations of our CCD reconstruction are that global weathering changed little across the LPEE (possibility 2), and/or that an increase in global weathering was accommodated by excess shelf carbonate burial (possibility 3). Proxy evidence for changes in weathering leading into the early Eocene hothouse is mixed. Lithium isotopes were relatively stable across the late Paleocene to early Eocene, interpreted as a tectonic limitation of weathering (Froelich & Misra, 2014; Misra & Froelich, 2012) or changes in the global denudation regime (Li & West, 2014). The Sr isotope trend through this interval likely reflects the emplacement of the North Atlantic Igneous Province (Hodell et al., 2007), which may have obscured the influence of continental weathering signals. If weathering did not appreciably increase in response to global temperature, it would suggest that our current parameterizations for a temperature‐dependent weathering feedback are incorrect. As this is a key process for returning long‐term CO_2_ to steady state, this possibility warrants further proxy investigation. Regarding possibility 3, we do not attempt to model changes in shelf burial in cGENIE. Rather, we deliberately restrict carbonate preservation and burial to ocean locations where the sediments lie deeper than 175 m to avoid environments where sediment porewaters at relatively shallow depths may become sulfidic and hence influence carbonate preservation via processes that are not currently represented in our sediment model. The global weathering flux in our model is therefore implicitly global weathering less an invariant (unspecified) carbonate removal term on continental shelves.

Experiments in ensemble 4 (Table S22), in which the global weathering rate is invariant, tested whether our data are consistent with (a) a progressively warming world without a weathering feedback or (b) increased weathering accommodated by increased burial on shelves. Higher *p*CO_2_ and temperature (without increased weathering flux) result in a noticeably shallower CCD (Table S22) at steady state. This is inconsistent with our global reconstruction and all previous regional studies (Hancock et al., 2007; Leon‐Rodriguez & Dickens, 2010; Slotnick et al., 2015) with the possible exception of Pälike et al. (2012), who interpreted that the CCD shallowed from late Paleocene to early Eocene. However, this interpretation compares late Paleocene off‐equatorial sites (Figure 2, dotted red line) to early Eocene equatorial sites (Figure 2, solid red line). Similarly, if excess weathering was accommodated by increased carbonate burial on shelves (a process not explicitly represented in our experiments), the CCD would shoal in response to higher *p*CO_2_ and temperature. We conclude that LPEE scenarios in which temperature and *p*CO_2_ are decoupled from global weathering, or in which a global weathering increase is entirely accommodated by increased shelf burial, are unlikely because of the lack of evidence for any substantial CCD shoaling.

One remaining question is whether in the simulation and subsequent calculation of the mean global CCD in cGENIE, the relatively coarse vertical discretization of ocean model grid (Figure S8) could lead to biases in our mean global CCD derivation and specifically, whether the CCD could get “pinned” in any way to a depth boundary between two different ocean grid levels, leading to an artificially low sensitivity of the CCD to changes in outgassing. Ensemble 5, with a smoother sediment model depth grid (Figure S9), explicitly tested for this possibility. We find that the smoother sediment model depth grid leads to a difference of no more than 30 m in the inferred CCD change between the extremes of outgassing rates (×0.6 and ×2.0) compared to the default (used in ensemble 1 experiments). In other words, we can rule out an artifact in our CCD‐identifying algorithm arising from the discretized‐in‐depth nature of the ocean circulation model grid. The modification of the seafloor depth at the data locations of Panchuk et al. (2008) did not affect the sensitivity of the steady‐state position of the CCD to changing rates of outgassing.

## 4 Conclusions

Our comprehensive data‐based CCD reconstruction from 75 DSDP/ODP/IODP deep‐sea drilling sites reveals a relatively stable CCD between the late Paleocene and the early Eocene (58 to 49 Ma) despite a warming of >3 °C (Cramer et al., 2009). Our modeling experiments demonstrate that significantly increased rates of global weathering are, counterintuitively, consistent with a relatively unresponsive CCD. The CCD can become decoupled not only from the background climate state, but also from total global carbonate burial fluxes, as burial of excess CaCO_3_ is accommodated at depths above the CCD. Driven only by changes in volcanic outgassing, the model is able to simultaneously reconcile observations of global ocean temperature and the CCD. However, the model underestimates the observed change in benthic δ^13^C across the LPEE. We attribute this model‐data mismatch to the lack of organic matter burial in our model. We suggest that Earth system changes across the LPEE were most likely driven by an increase in global volcanic outgassing rates, but models that include feedbacks in both inorganic and organic carbon burial will be necessary to simultaneously reproduce all the observed trends.

Our findings are not specific to the question of the drivers of LPEE climate, but create an unexpected challenge to the use of CCD reconstructions to constrain global carbon cycling over long timescales for any interval in Earth. First, further constraints about carbonate accumulation at shallower depths (e.g., large global compilations of carbonate abundance like this one and/or carbonate accumulation rates; Archer, 1996; Catubig et al., 1998; Pälike et al., 2012) in conjunction with numerical modeling are needed if CCD reconstructions are to be interpreted correctly. Second, we question whether numerical models that omit organic matter oxidation and the consequent release of CO_2_ to pore waters are fully appropriate for using CCD changes (or CCD stasis) as a proxy constraint.

## Supporting information

Supporting Information S1

Supporting Information S2

Data Set S1

Data Set S2

Data Set S3
