# Supplementary material for: Early Cenozoic Decoupling of Climate and Carbonate Compensation Depth Trends
Source: Paleoceanogr Paleoclimatol. 2019 Jun 17;34(6):930–45. doi: 10.1029/2019PA003601 (PMC6774345; doi:10.1029/2019PA003601)
Supplement: Supplementary file 1 — Supporting Information S1 [file PALO-34-930-s001.docx]

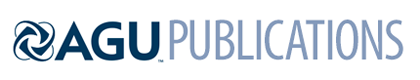


*Paleoceanography and Paleoclimatology*

Supporting Information for

**Early Cenozoic Decoupling of Climate and Carbonate Compensation Depth Trends**

S.E. Greene^1^, A. Ridgwell^2,3^, S. Kirtland Turner^3^, D.N. Schmidt^4^, H. Pälike^5^, E. Thomas^6,7^, L.K. Greene^8,9^, and B.A.A. Hoogakker^10^

^1^School of Geography, Earth and Environmental Sciences, University of Birmingham, Birmingham, UK

^2^BRIDGE, School of Geographical Sciences, University of Bristol, Bristol, UK

^3^Department of Earth Sciences, University of California at Riverside, Riverside, CA, USA

^4^School of Earth Sciences, University of Bristol, Bristol, UK

^5^MARUM-Center for Marine Environmental Sciences, University of Bremen, Bremen, Germany

^6^Department of Geology and Geophysics, Yale University, New Haven, CT, USA

^7^Department of Earth and Environmental Sciences, Wesleyan University, Middletown, CT, USA

^8^University Program in Ecology, Duke University, Durham, NC, USA

^9^ Department of Evolutionary Anthropology, Duke University, Durham, NC, USA

^10^ Institute of Life and Earth Sciences, Heriot Watt University, Edinburgh, UK

**Contents of this file**

Text S1 to S2

Figures S1 to S9

Table Captions S1-S22

Matlab Script Captions S1-S2

**Additional Supporting Information (Files uploaded separately)**

Tables S1-S22: PP2019.Greeneetal.SupplementaryTables.xlsx

Matlab Scripts S1-S2: PP2019_Greeneetal_Subsidence.m; PP2019_Greeneetal_plot_CCDcontour.m

Code availability statements (muffin.pdf or muffin.README.tex)

**Introduction**

The supplementary information includes text describing paleodepth calculation and error (Text S1) and how to obtain model code and supporting files (Text S2), supplemental figures S1-S9, and captions for both supplemental Tables S1-S22 and two supplemental Matlab Scripts. An excel spreadsheet (Tables S1-S22) and the supplemental Matlab Scrips (S1-S2) are provided as separate files.

Text S1.

**Paleodepth/Estimates of paleodepth error**

Paleodepth computed following Cramer et al.[2009], but with a simplified sediment unloading term. (Supplemental script for computing paleodepth is provided as a separate matlab script - see section entitled **‘Supplemental matlab scripts’** below).

$${Depth}_{t}={Depth}_{i}+S\left( {Age}_{c}-t \right)+0.66({Depth}_{c}-{SedThickness}_{t})$$

where

$${Depth}_{i}={Depth}_{c}-S\left( {Age}_{c} \right)-0.66*SedThickness$$

and

$${SedThickness}_{t}=\frac{SedThickness}{{Age}_{c}}*t$$

and

$$S\left( x \right)=\left\{ \begin{aligned} 365\sqrt{x} for {Age}_{c}<20Ma \\ 3051(1-\frac{8}{\pi^{2}}e^{-0.0278x} for {Age}_{c}\geq20Ma \end{aligned} \right.$$

*Depth_t_* = Paleodepth at time (t) in the past

*Depth_i_* = Initial Depth of basement at time Age_c_

*Depth_c_* = Depth of crust below seafloor

*SedThickness* = Total sediment cover

*Sedthickness_t_* = Total sediment cover at time t

*Age_c_* = Crust age

*Age_t_* = Age

*t* = Time in the past (Ma)

Errors associated with paleodepth calculation are highly variable from site to site. Broadly, these errors can be separated into two types. First, and more easily quantifiable, is the error associated with the site-specific variables in the subsidence equations, namely basement age and sediment cover. Errors in basement age differ greatly between regions. For most localities (where seafloor anomaly patterns are clear and well described), the error is likely small (<5 Ma) but it could be upwards of 10 Ma, particularly in swaths of the tropical Pacific [*Müller et al.*, 2008]. However, hot, young ocean crust cools and subsides much faster than older, colder crust, so that the error in seafloor age propagates to paleodepth errors in a highly non-linear manner. A 5 Ma error in seafloor age will result in a much larger error in paleodepth for a site that was underlain by fresh crust at the time of reconstruction vs. one situated on older crust. The errors associated with sediment cover are likely to be negligible in most cases. Sediments are always less dense than underlying basement, therefore each meter of sediment cover depresses the underlying crust by less than a meter. Only sediment cover errors on the order of hundreds of meters to kilometers can result in paleodepth errors of hundreds of meters. Similarly, the linear sedimentation rate assumption that we use can only result in paleodepth errors of hundreds of meters if the error in the calculated thickness of sediment cover at the time of paleodepth reconstruction is of similar magnitude.

The second type of error arises from the degree to which the geological histories of individual sites deviate from the mean empirically derived subsidence curve, i.e., are not situated on ‘normal’ oceanic crust generated at a mid-ocean spreading center. Some sites may have experienced uplift due to local tectonic factors or simply may have an atypical underlying crustal origin which affects the crustal cooling curve trajectory (e.g. Demerara Rise, Ontong-Java Plateau, Walvis Ridge, Shatsky Rise). Although difficult to quantify, this type of error is most likely the largest source of paleodepth error for most sites, with many sites drilled on such atypical crust.

Text S2.

**Model code and availability of supporting files**

*The source code of the cGENIE Earth system model used here—together with specific experimental configurations, boundary conditions, and data-forcing files—is available for download on GitHub. See accompanying code availability statement (muffin.pdf or muffin.README.tex).*

**Supplemental Figures:**


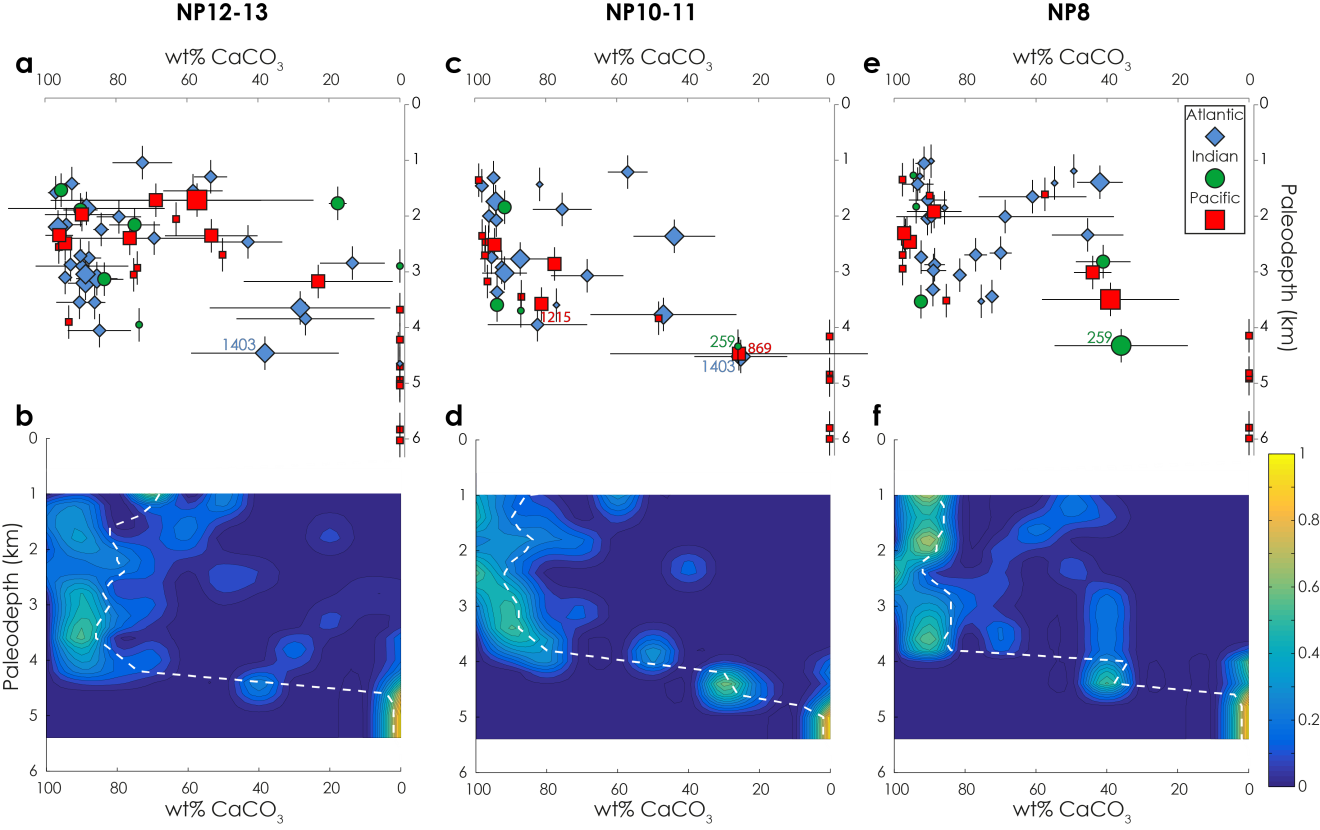


**Fig. S1. Reconstructed CCD across the LPEE. a,c,e)** Paleodepth vs. wt% CaCO_3_ for three time-slices spanning the LPEE. Symbol size denotes the number of wt% CaCO_3_ measurements averaged for each site (small: 1 measurement, medium: 2-9 measurements, large: ≥10 measurements). Horizontal error bars represent 1σ from the mean wt% CaCO_3_ recorded at each site within the time-slice. Vertical error bars represent ±300m paleodepth (an estimate derived by trebling the paleodepth error estimate for sites <25Ma [*Sclater et al.*, 1985] or doubling the ±150m suggested by Van Andel [1975] and Van Andel et al. [1975] for sites underlain by crust older than a few million years at the time of paleodepth reconstruction). **b,d, f)** Same dataset as above, but contoured and normalized to highlight where the data at any given depth fall in wt% CaCO_3_ space (see Methods). The white dashed lines denote the median wt% value with 50% of the data at higher or lower wt% values for any given depth. Color represents ‘density’ of normalized weight, and sums to 1 at any given depth.


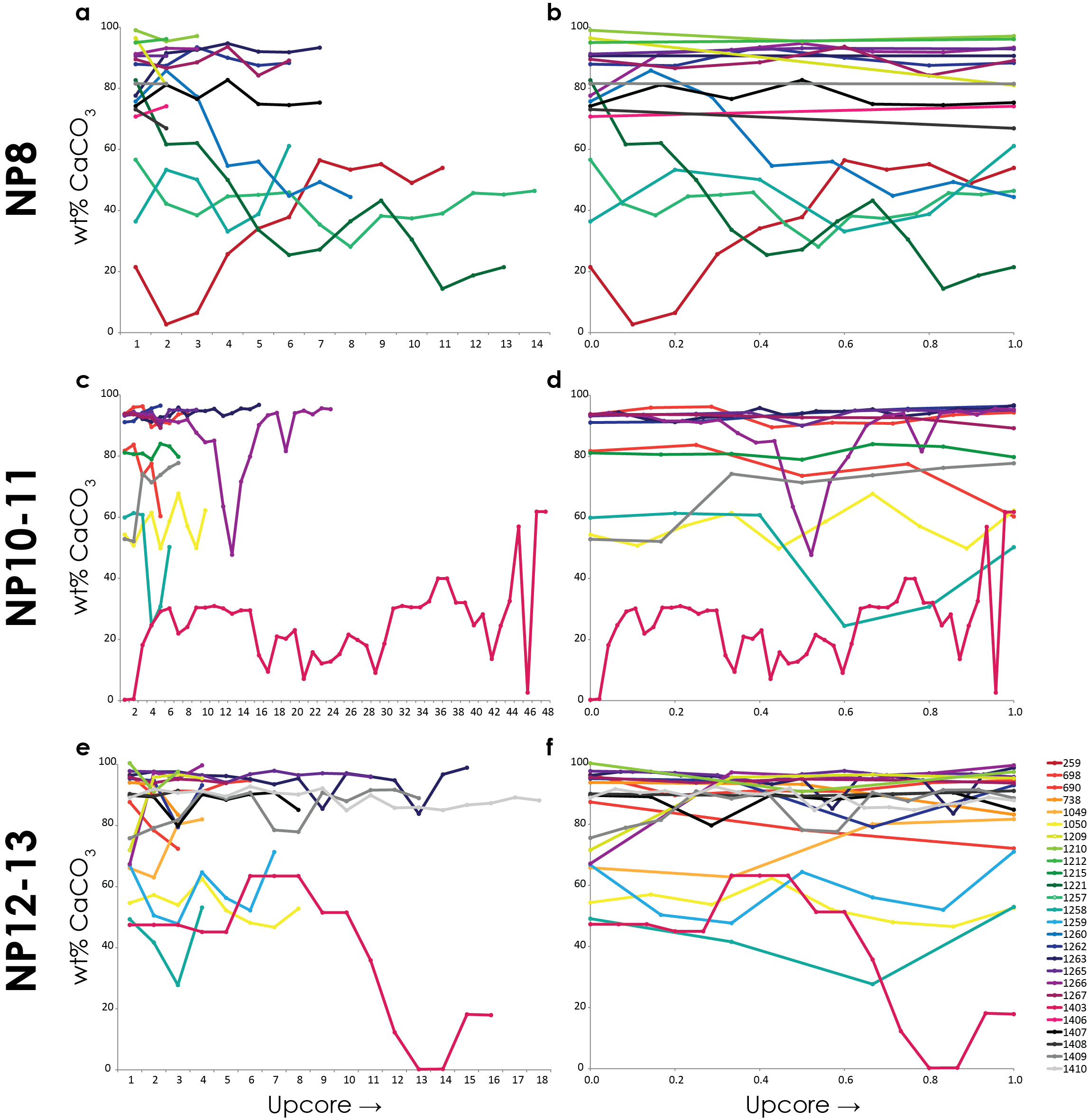


**Fig. S2. Temporal evolution of wt% CaCO_3_ in each time-slice for sites with multiple measurements in that time-slice**. Because most of these sites the lack detailed age models required to align the individual CaCO_3_ wt% measurements, we plot the data in two ways. At left, (panels **a**,**c**,and **e**) the wt% CaCO_3_ measurements for individual sites are plotted in stratigraphic order with the basal (oldest) measurement on the left. At right (panels **b**, **d**, and **f**), the data are scaled horizontally to align the lowermost and uppermost stratigraphic samples for each site.


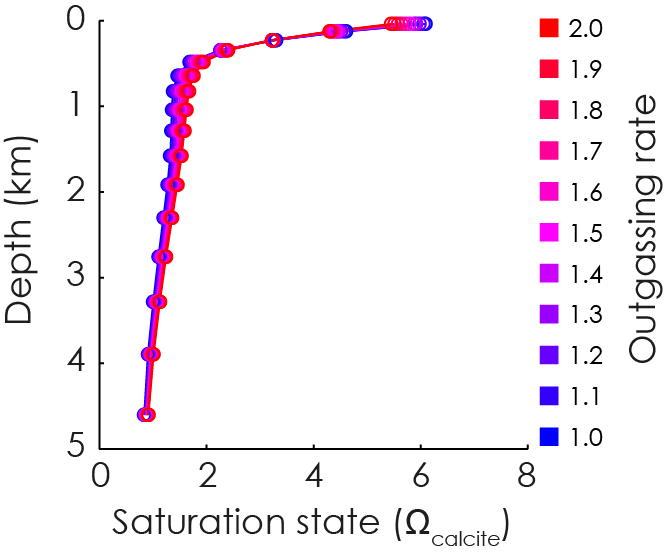


**Fig. S3.** **Water column depth vs. mean calcite saturation state for members of ensemble 1.**


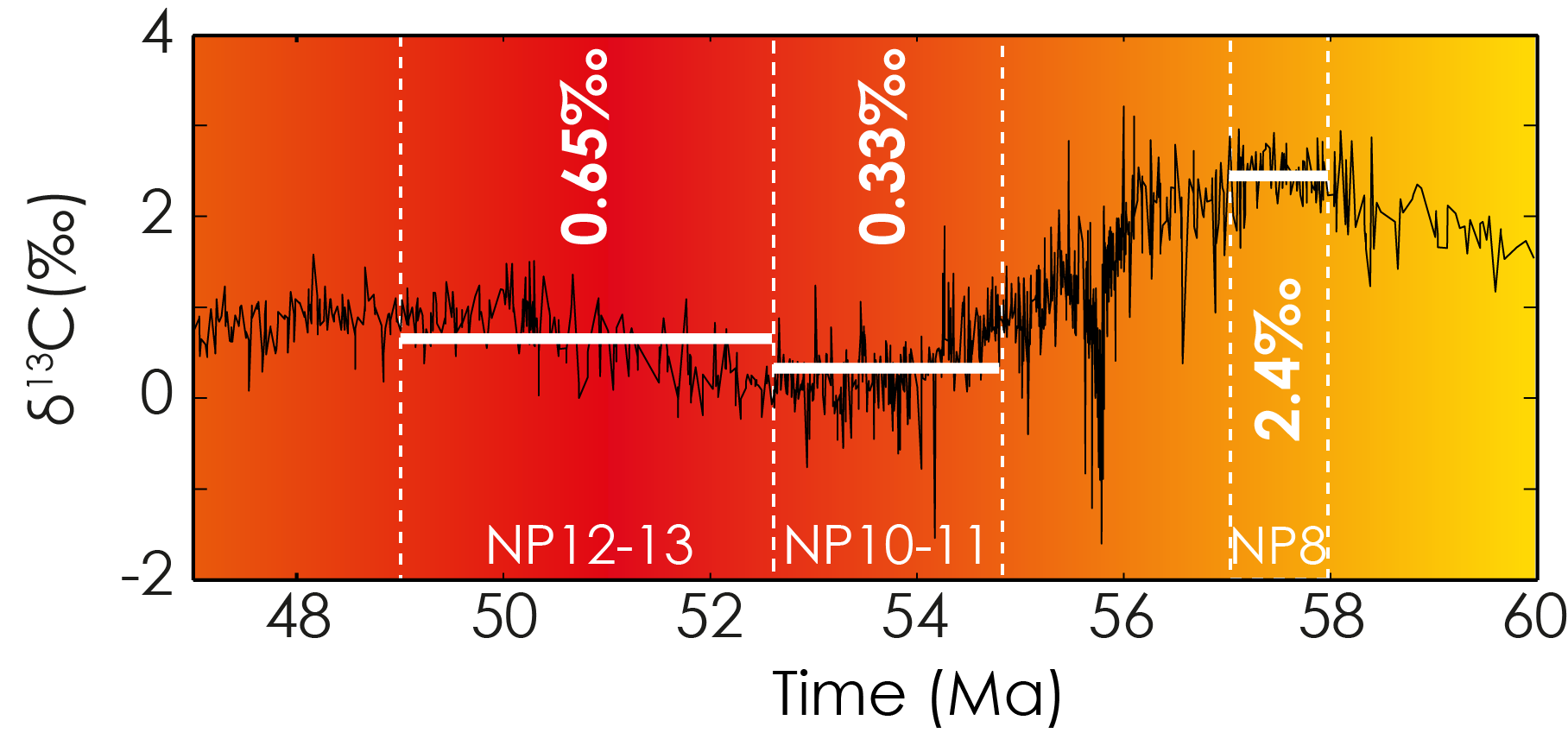


**Fig. S4**. **LPEE benthic foraminiferal isotopic record of δ^13^C.** Data from Cramer et al. [2009]**.** The three time-slices are demarcated by their respective nannofossil zonations. Mean δ^13^C (white lines) are given for each time-slice.


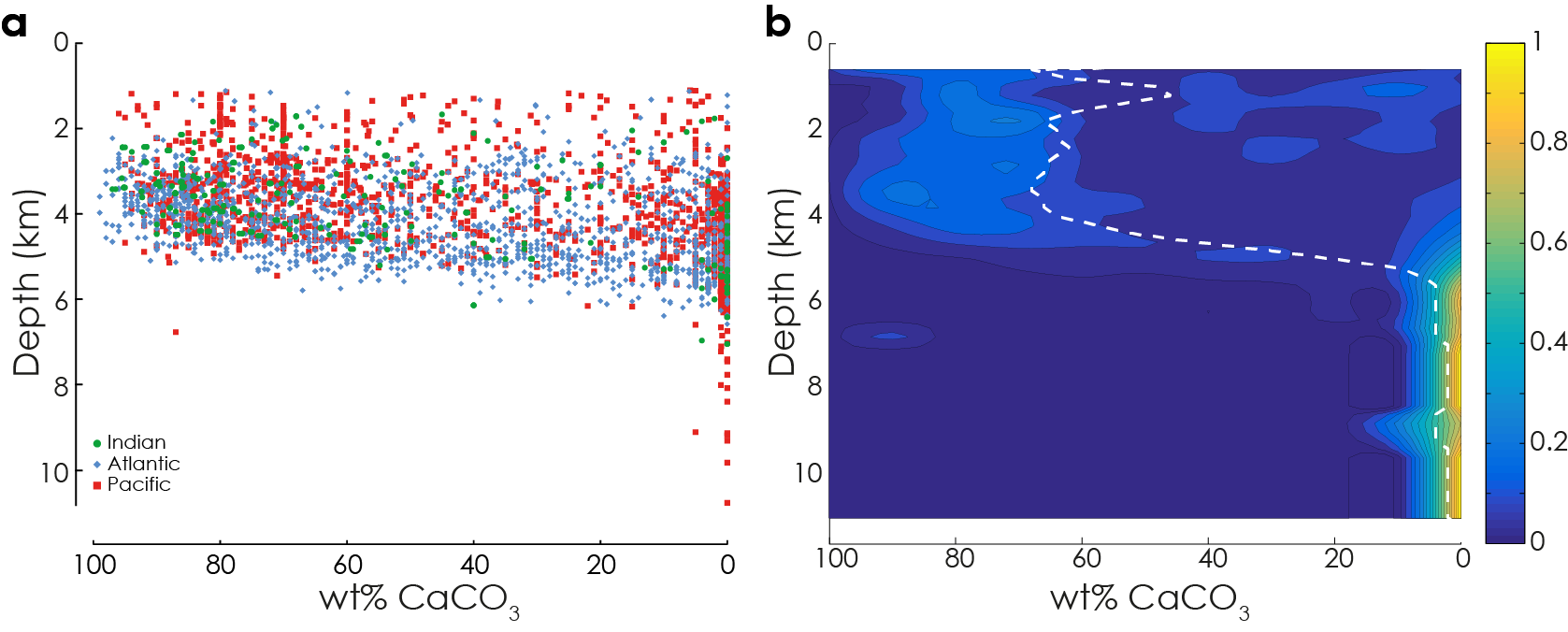


**Fig. S5. Modern coretop wt% CaCO_3_ and the average global CCD.** a) Modern coretop wt% CaCO_3_ for sites ≥1km depth from Archer [1996] (Southern Ocean excluded). b) Data in panel A contoured and normalized as in Figs. 3, 5 (See Methods).


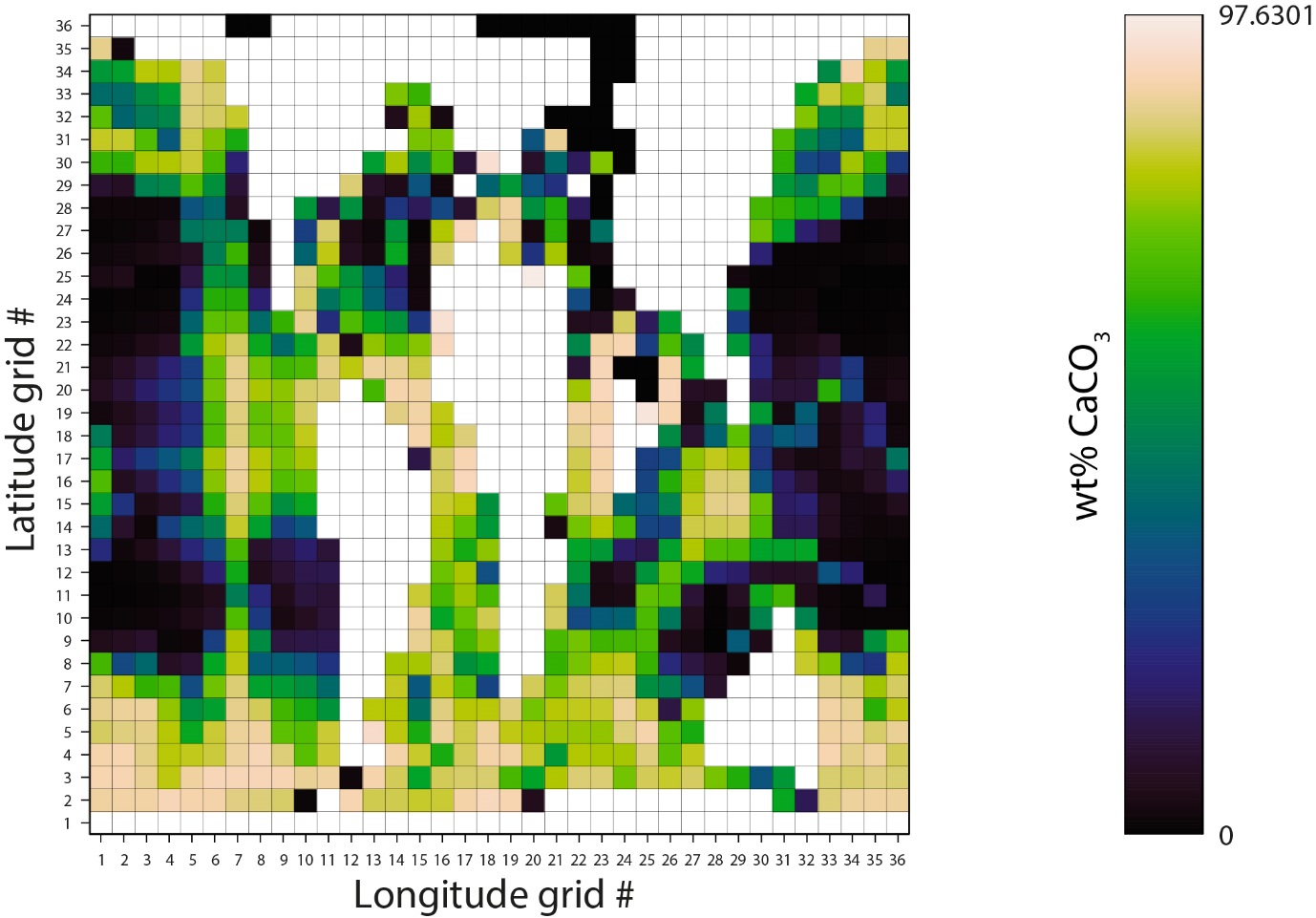


**Fig. S6. Example coretop wt% CaCO_3_ output from cGENIE.**


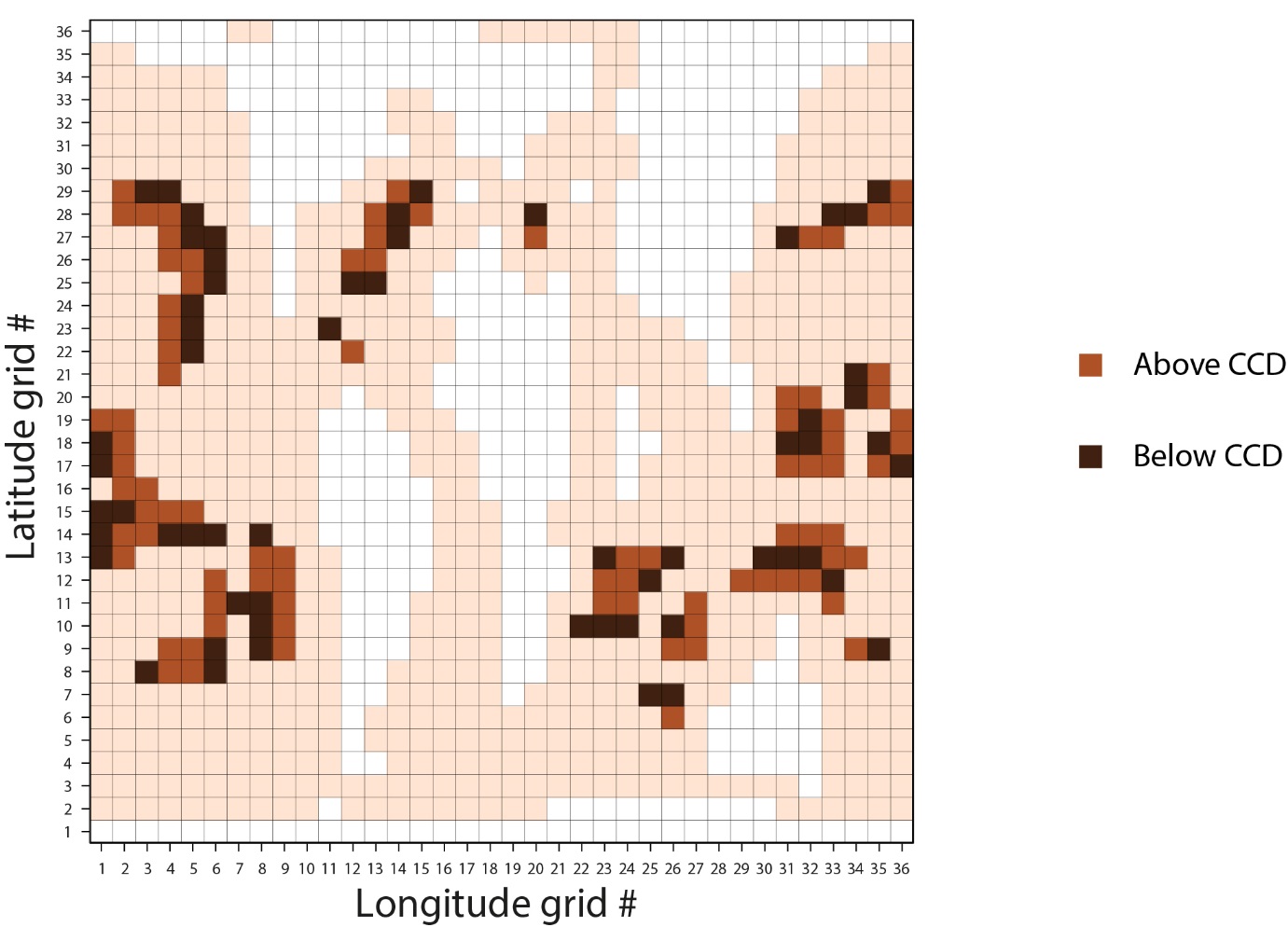


**Fig. S7. CCD-spanning grid point pairs used in CCD extraction.**

**
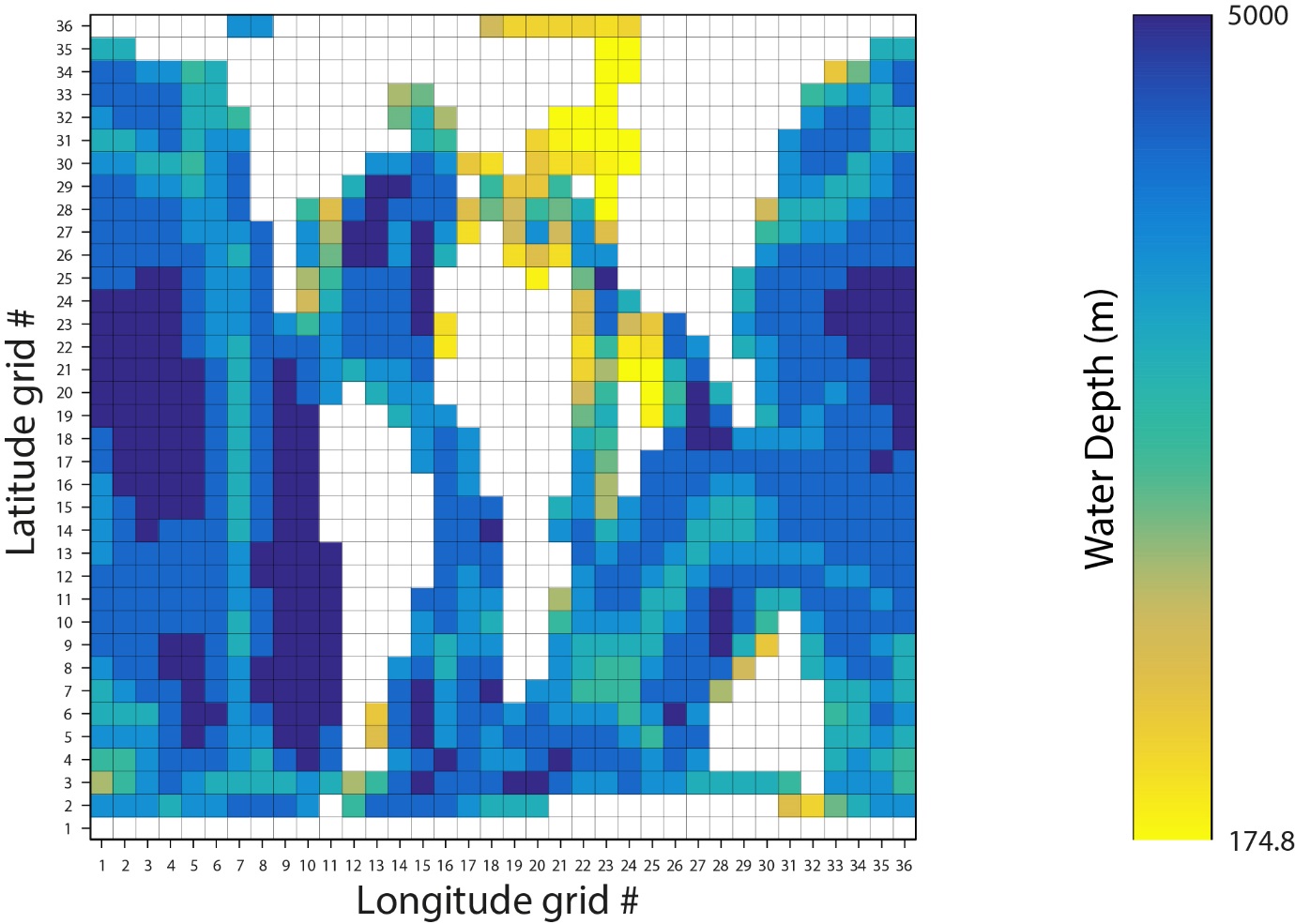
**

**Fig. S8. Standard sediment model depth grid discretized at 16 ocean levels.**

**
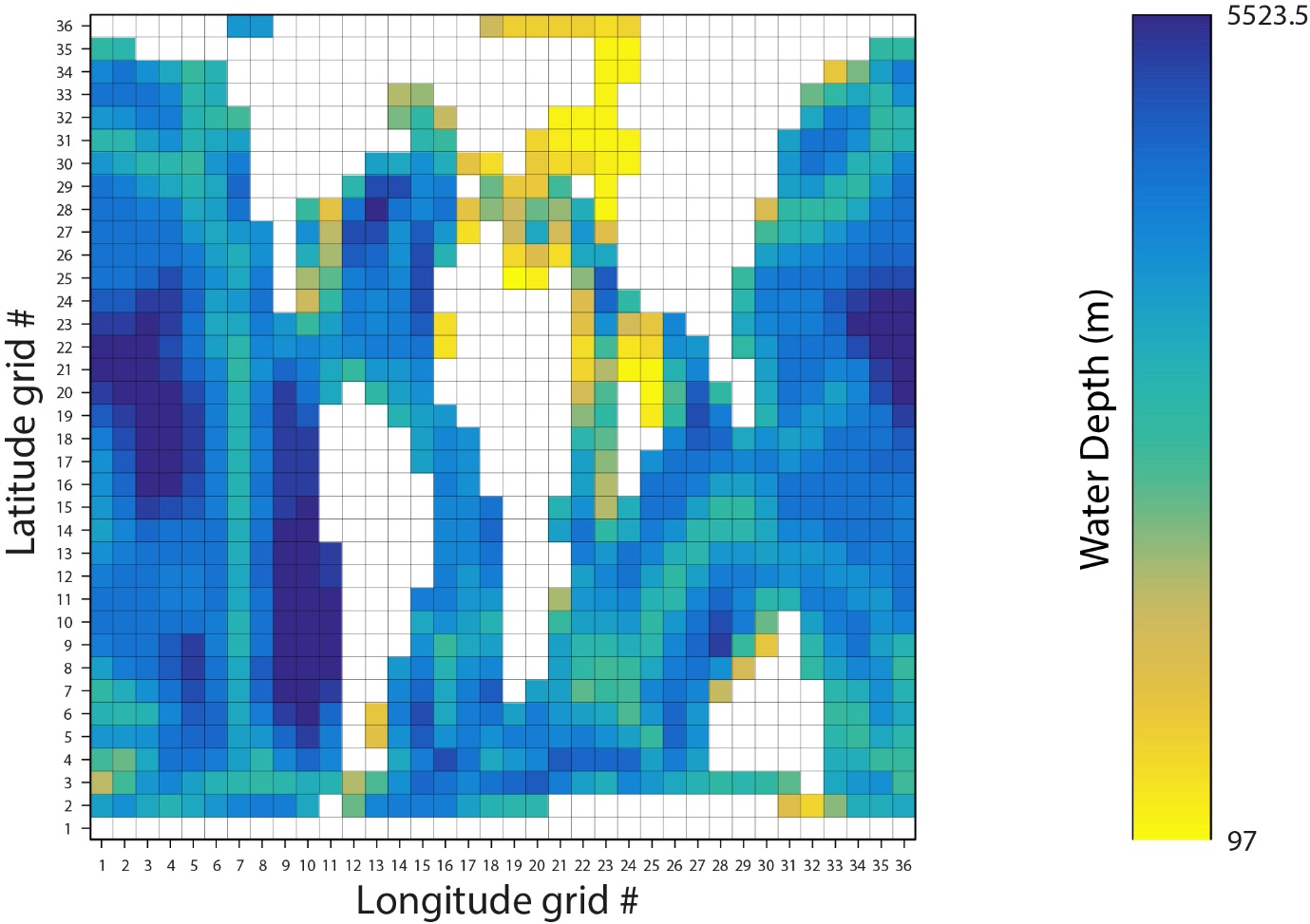
**

**Fig. S9**. **Alternative sediment model depth grid discretized at 64 levels and with a maximum 6000 m (rather than 5000 m) ocean depth.**

**Supplemental tables**

Supplemental tables can be found in accompanying excel file (PP2019.Greeneetal.SupplementaryTables.xlsx).

**Tables S1-S3.** **Raw data for CCD time-slice reconstruction for NP8, NP10-11, and NP12-13, respectively.** Raw data for each CCD time-slice reconstruction (program, site number, current and paleo- latitude/longitude, underlying basement age, current water depth, sediment cover, reconstructed paleodepth (see Methods), wt% CaCO_3_ mean, and individual wt% CaCO_3_ measurements.

**Tables S4-S18**. **Model output: time series of *p*CO_2_ and Ca^2+^ weathering flux for each ensemble #1 experiment.**

**Tables S19-S22.** **Final year model output for each experiment in each ensembles 1-4.** Final year model output for each experiment ensembles 1-4 (outgassing rate modification factor relative to x3 pre-industrial *p*CO_2_, *p*CO_2_, mean ocean [DIC], mean ocean [ALK], POC export, Ca^2+^ weathering flux, mean ocean temperature, mean land surface air temperature, overturning stream function min/max, and final year CSH and CCD (following Goodwin and Ridgwell [2010]). For ensembles 2-4 bolded columns indicate model variables roughly fixed across all experiments within the ensemble.

**Supplemental Matlab Scripts**

Two supplemental matlab scripts are provided.

**Matlab Script S1.** To compute paleodepth following Cramer et al.[2009]: PP2019_Greeneetal_Subsidence.m

**Matlab Script S2.** For contour plotting carbonate wt% vs. (paleo)depth (e.g. Figs. 4, 6, S5): PP2019_Greeneetal_plot_CCDcontour.m.

**References**

Archer, D. E. (1996), An atlas of the distribution of calcium carbonate in sediments of the deep sea, *Global Biogeochemical Cycles*, *10*(1), 159-174.

Cramer, B. S., J. R. Toggweiler, J. D. Wright, M. E. Katz, and K. G. Miller (2009), Ocean overturning since the Late Cretaceous: Inferences from a new benthic foraminiferal isotope compilation, *Paleoceanography*, *24*(4), PA4216.

Goodwin, P., and A. Ridgwell (2010), Ocean-atmosphere partitioning of anthropogenic carbon dioxide on multimillennial timescales, *Global Biogeochemical Cycles*, *24*(2), GB2014.

Müller, R. D., M. Sdrolias, C. Gaina, and W. R. Roest (2008), Age, spreading rates, and spreading asymmetry of the world's ocean crust, *Geochemistry, Geophysics, Geosystems*, *9*(4), Q04006.

Sclater, J. G., L. Meinke, A. Bennett, and C. Murphy (1985), The depth of the ocean through the Neogene, *Geological Society of America Memoirs*, *163*, 1-20.

Van Andel, T. H. (1975), Mesozoic/cenozoic calcite compensation depth and the global distribution of calcareous sediments, *Earth and Planetary Science Letters*, *26*(2), 187-194.

Van Andel, T. H., G. R. Heath, and T. C. Moore (1975), Cenozoic History and Paleoceanography of the Central Equatorial Pacific Ocean: A Regional Synthesis of Deep Sea Drilling Project Data, *Geological Society of America Memoirs*, *143*, 1-223.
