# Supplementary material for: Early Cenozoic Decoupling of Climate and Carbonate Compensation Depth Trends
Source: Paleoceanogr Paleoclimatol. 2019 Jun 17;34(6):930–45. doi: 10.1029/2019PA003601 (PMC6774345; doi:10.1029/2019PA003601)
Supplement: Supplementary file 2 — Supporting Information S2 [file PALO-34-930-s002.pdf]

Code availability statement the cGENIE.muffin model

Andy Ridgwell

March 1, 2019

muffin overview

The code for the cGENIE.muffin model is hosted on GitHub. The current version can be obtained by cloning:

https://github.com/derpycode/cgenie.muffin

A manual, detailing code installation, basic model configuration, plus an extensive series of tutorials covering various aspects of muffin capability, experimental design, and results output and processing, is provided. A PDF of the manual can be downloaded here:

http://www.seao2.info/cgenie/docs/muffin.pdf

The latex source and most up-to-date built PDF file can be obtained by cloning:

https://github.com/derpycode/muffindoc

Instructions summary

The muffin manual contains instructions for obtaining, installing, and testing the code, plus how to run experiments. Specifically:

Section 1.1 – provides a basic over-view of the software environment required for installing and running muffin.

Section 1.2.2 – provides a basic over-view of cloning and testing the code.

Secion 15.4 – provides a detailed guide to cloning the code and configuring a Ubuntu (18.04) software environment including netCDF library installation, plus running a basic test.

Secion 15.6 – provides a detailed guide to cloning the code and configuring a macOS software environment including netCDF library installation, plus running a basic test.

Secion 1.3 – provides a basic guide to running experiments (also see 1.6 and 1.7).

Secion 1.4 – provides a basic introduction to model output (much more detail is given in Section 12).

Model experiments

Configuration files for the specific experiments presented in the paper can be found in the directory:

`cgenie.muffin\genie-userconfigs\MS\greeneetal.2019`

Details of the different experiments, plus the command line needed to run each one, are given in `readme`.txt.
